# Khz-cp (crude polysaccharide extract obtained from the fusion of *Ganoderma lucidum* and *Polyporus umbellatus* mycelia) induces apoptosis by increasing intracellular calcium levels and activating P38 and NADPH oxidase-dependent generation of reactive oxygen species in SNU-1 cells

**DOI:** 10.1186/1472-6882-14-236

**Published:** 2014-07-10

**Authors:** Tae Hwan Kim, Ju Sung Kim, Zoo Haye Kim, Ren Bin Huang, Young Lye Chae, Ren Sheng Wang

**Affiliations:** 1 Department of Radiotherapy, The First Affiliated Hospital, Guangxi Medical University, Nanning, China; 2 Clinical Medicine, Harbin Medical University, Harbin, China; 3 Graduate School of Information Science, Nagoya University, Nagoya, Japan; 4 Department of Pharmacology, Guangxi Medical University, Nanning, China; 5 Korea Institute of Science and Management Career College, Seoul, South Korea

## Abstract

**Background:**

Khz-cp is a crude polysaccharide extract that is obtained after nuclear fusion in *Ganoderma lucidum* and *Polyporus umbellatus* mycelia (Khz). It inhibits the growth of cancer cells.

**Methods:**

Khz-cp was extracted by solvent extraction. The anti-proliferative activity of Khz-cp was confirmed by using Annexin-V/PI-flow cytometry analysis. Intracellular calcium increase and measurement of intracellular reactive oxygen species (ROS) were performed by using flow cytometry and inverted microscope. SNU-1 cells were treated with p38, Bcl-2 and Nox family siRNA. siRNA transfected cells was employed to investigate the expression of apoptotic, growth and survival genes in SNU-1 cells. Western blot analysis was performed to confirm the expression of the genes.

**Results:**

In the present study, Khz-cp induced apoptosis preferentially in transformed cells and had only minimal effects on non-transformed cells. Furthermore, Khz-cp was found to induce apoptosis by increasing the intracellular Ca^2+^ concentration ([Ca^2+^]_*i*_) and activating P38 to generate reactive oxygen species (ROS) *via* NADPH oxidase and the mitochondria. Khz-cp-induced apoptosis was caspase dependent and occurred *via* a mitochondrial pathway. ROS generation by NADPH oxidase was critical for Khz-cp-induced apoptosis, and although mitochondrial ROS production was also required, it appeared to occur secondary to ROS generation by NADPH oxidase. Activation of NADPH oxidase was shown by the translocation of the regulatory subunits p47^phox^ and p67^phox^ to the cell membrane and was necessary for ROS generation by Khz-cp. Khz-cp triggered a rapid and sustained increase in [Ca^2+^]_*i*_ that activated P38. P38 was considered to play a key role in the activation of NADPH oxidase because inhibition of its expression or activity abrogated membrane translocation of the p47^phox^ and p67^phox^ subunits and ROS generation.

**Conclusions:**

In summary, these data indicate that Khz-cp preferentially induces apoptosis in cancer cells and that the signaling mechanisms involve an increase in [Ca^2+^]_*i*_, P38 activation, and ROS generation *via* NADPH oxidase and mitochondria.

## Background

Cancer develops because of abnormal cellular proliferation or defective apoptosis that leads to uncontrolled growth [1]. Therefore, new treatments that target the proliferation and apoptosis of cancer cells are necessary. Under normal conditions, programmed cell death occurs after exposure to pathological factors. Apoptosis involves cell shrinkage, condensation of nuclei and chromatin, and DNA fragmentation, all of which result in unmistakable cellular morphology. Apoptosis is initiated by external signals through a series of cysteine acid proteases, including important regulatory factors such as caspases. Cytochrome c–mediated Casp3 activation may be utilized by a specific and restricted set of external apoptosis stimuli. Defective signaling during the regulation of cell death can result in the abnormal proliferation of cells and can cause cancer. Therefore, repairing defective cell death mechanisms or developing drugs or food components that induce cell differentiation may be a promising approach for the generation of anticancer agents [2,3]. In particular, many studies are being performed to identify natural products that can be used as anticancer drugs and that do not have the toxicity and adverse effects associated with chemotherapeutic drugs. Several biologically active ingredients that show effective anticancer activity have been derived from edible or medicinal mushrooms [4-6], and the anticancer effects of *Ganoderma lucidum* have been described in various studies [7-10]. Additionally, *Polyporus umbellatus* induces G2/M cell cycle arrest and apoptosis in HepG2 cells, thereby causing growth suppression [11].

**Figure 1:**
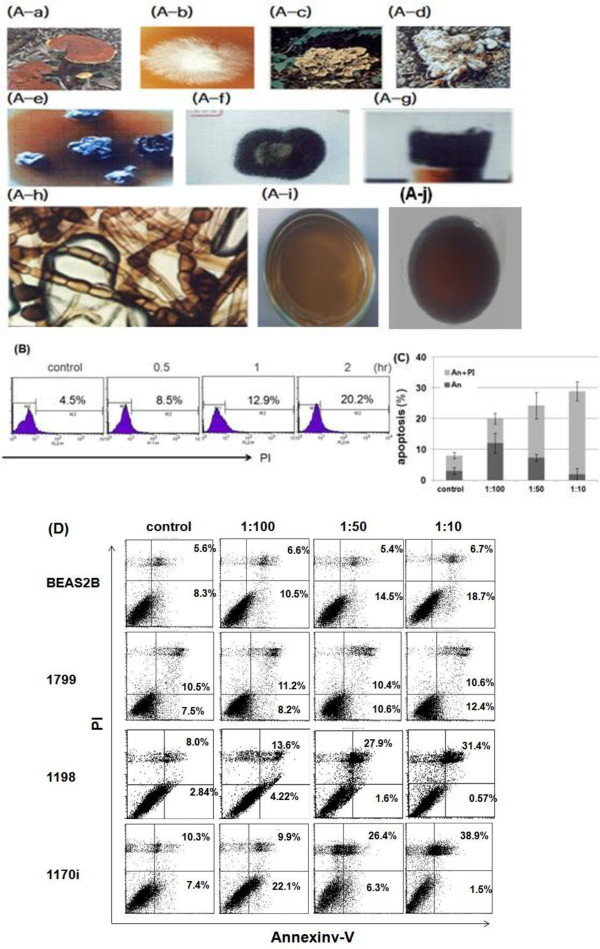
**Khz-cp induces apoptosis in transformed cells. (A)** (A-a) The shape and type of fused fruiting bodies. (A-b) Hyphae isolated from a *Ganoderma lucidum* mushroom on a petri dish. (A-c) Shape of *G. lucidum*. (A-d) Shape and type of fused fruiting bodies and hyphae from *Polyporus umbellatus*. (A-e) Fusion of *G. lucidum* and *P. umbellatus*. (A-f) The fused hyphae of *G. lucidum* and *P. umbellatus*. (A-g) Agar-cultured fusion fungi. (A-h) DNA from fused hyphae (Khz). (A-i) Cultivation conditions for Khz. (A-j) Khz crude polysaccharides (Khz-cp). **(B)** Analysis of apoptosis using propidium iodide (PI) staining. SNU-1 cells were treated with a 1:100 dilution of Khz-cp, and apoptosis was analyzed after 0.5, 1, and 2 h by flow cytometry. The data provided are representative of more than 3 experiments. **(C)** SNU-1 cells were treated 1 h with a 1:100 dilution of Khz-cp and stained with An and PI for flow cytometric analysis. The data represent the mean ± SD values. **(D)** BEAS-2B, 1799, 1198, and 1170-I cells were treated with Khz-cp (1:100 dilution, 1 h), and apoptosis was examined by annexin-V-FITC (An) and PI staining followed by flow cytometric analysis. The data are representative of more than 3 experiments.

Khz-cp is an extract mixture from the mycelia of a G. lucidum and P. umbellatus nuclear fusion (Figure 1A). The anticancer effect of the fusion of G. lucidum and P. umbellatus has been previously demonstrated [12,13]. In this study, we investigated the mechanism underlying Khz-induced cell death in gastric cells.

Oxidative stress is widely implicated in apoptotic and non-apoptotic cell death [14-16]. The major sources of intracellular reactive oxygen species (ROS) include NADPH oxidase and the mitochondrial electron transport chain (ETC.). When ROS production, either by the mitochondria or by NADPH oxidase, becomes excessive, the natural cellular antioxidant defense system is overwhelmed, which results in oxidative stress. Cancer cells are more susceptible to oxidative stress than healthy cells, and some anticancer agents such as cisplatin, arsenic trioxide (As_2_O_3_), and 2-methoxyestradiol exert their effects by inducing ROS production [17-19]. Much of the available data indicate that the ROS that accumulate during cell death are generated by the mitochondria in response to impairment of the mitochondrial respiratory chain [20-23]. Although mitochondrial ROS production is regarded as an integral component of the apoptotic program, the role of NADPH oxidase as a primary source of ROS during the induction of apoptosis has also been reported [24-26]. Sustained elevation of the intracellular Ca^2+^ concentration ([Ca^2+^]_*i*_) is associated with the induction of apoptosis [27]. When the cytoplasmic [Ca^2+^]_*i*_ increases, the mitochondria take up Ca^2+^ and function as a Ca^2+^ buffer; however, excessive accumulation of mitochondrial Ca^2+^ triggers apoptosis, at least in part by inducing ROS generation *via* the mitochondrial ETC. An increase in cytoplasmic [Ca^2+^]_*i*_ can also activate NADPH oxidase, which has been well documented in neutrophils [28]. In some cell types, the activation of protein kinase C *via* intracellular Ca^2+^ leads to the phosphorylation of the p47^phox^ subunit and subsequent enzyme assembly [29].

In the present study, we investigated the role of Khz-cp in cellular apoptosis and found that Khz-cp induced a sustained increase in [Ca^2+^]_*i*_ that resulted in ROS generation by NADPH oxidase *via* P38 and, finally, cellular apoptosis.

## Methods

### Cell lines and Khz-cp treatment

The BEAS-2B (normal immortalized), 1799 (non-transformed), 1198 (transformed but non-tumorigenic), and 1170-I (tumorigenic) cell lines that compose the *in vivo* lung carcinogenesis model used in this study have been previously described [30,31]. The human gastric cancer cell line SNU-1 was maintained in RPMI 1640 media supplemented with 10% fetal bovine serum, 100 U/ml penicillin G sodium, 100 μg/ml streptomycin sulfate, and 0.25 μg/ml amphotericin B. Unless otherwise indicated, all the cells were treated with Khz-cp diluted 1:100 in the media.

### Extraction of Khz-cp (crude polysaccharide extract obtained from the fusion of *G. lucidum* and *P. umbellatus* mycelia)

First, 1 kg of powder was added to 8.5 L of clean water, heated to 115°C, and extraction was performed for 60 min under pressure. This was followed by a 60-min maturation period. Next, the remaining water from the first extraction was added to 7.5 L of clean water and heated to 115°C; extraction was performed under pressure for 60 min, followed by maturation for a further 60 min. The first and second extracts were then mixed, boiled, and placed in bottles after 5 min.

We purified Khz-cp from Khz by using the Sevag method for deproteinization. The Sevag reagent, which is a 4:1 mixture of chloroform and n-butanol, was added with shaking; the volume of the reagent added was one-fourth that of the sample solution. After the mixture was allowed to stand and separate, the water layer and the solvent layer at the junction of the denatured protein were removed. This step was repeated several times until the denatured protein content was minimized. Preliminary experiments showed that a fourfold excess of 95% ethanol with respect to the sample volume was appropriate for precipitation. Therefore, after the removal of the denatured protein, 95% ethanol (4 times the sample volume) was added slowly until additional precipitation did not occur. The mixture was centrifuged and the supernatant was removed. The sediment collected was a brown precipitate and represented the total bacterial crude polysaccharide fraction. The polysaccharides were then dissolved and filtered by membrane ultrafiltration (molecular weight cutoff: 100,000 Da). Khz-cp was obtained from BrainGroup (Seoul, South Korea).

### Reagents and antibodies

Mitochondrion-targeted ubiquinone (MitoQ) is an ubiquinol antioxidant attached to a lipophilic triphenylphosphonium (TPP) cation [32]. MitoQ and TPP were kind gifts from Dr. Michael P. Murphy (Medical Research Council Dunn Human Nutrition Unit, UK). SB203580, apocynin, and cyclosporin A (CsA) were purchased from Calbiochem (San Diego, CA, USA), and *N*-acetyl cysteine (NAC) and ethylene glycol tetraacetic acid (EGTA) were purchased from Sigma (St. Louis, MO, USA). z-VAD-fmk was obtained from R&D Systems (Minneapolis, MN, USA), diphenylene iodonium (DPI) was from Cayman Chemical (Ann Arbor, MI, USA), and BAPTA-AM was from Invitrogen (Eugene, OR, USA).

Antibodies against p38 (sc-7972), p47^phox^ (sc-14015), p67^phox^ (sc-15342), caspase 3 (sc-7148), PARP (sc-7150), and cytochrome *c* (sc-13561) were purchased from Santa Cruz Biotechnology (Santa Cruz, CA, USA). Anti-p-P38 (9255) antibodies were obtained from Cell Signaling (Danvers, MA, USA), and a COX IV antibody (A21347) was obtained from Invitrogen.

### Western blot analysis

Cells were lysed in an extraction buffer (31.25 mM Tris–HCl [pH 6.8], 1% sodium dodecyl sulfate [SDS], 10% glycerol, and 2.5% mercaptoethanol), and the whole cell lysate was subjected to 10% SDS-polyacrylamide gel electrophoresis. Size-fractionated proteins on the gel were transferred onto a nitrocellulose membrane. The membrane was blocked in 5% skim milk in Tris-buffered saline containing 0.05% Tween 20 and was incubated with a primary antibody. After washing, the membrane was incubated with the peroxidase-conjugated secondary antibody. The protein band of interest was detected using enhanced chemiluminescence reagents (Amersham).

### Apoptosis assay

Cells treated with Khz-cp were washed twice in cold phosphate-buffered saline (PBS) and stained with annexin-V-FITC (A13199; Invitrogen) and propidium iodide (PI) according to the manufacturer’s instructions. Briefly, annexin-V-FITC (5 μL) was added to the cells, which were resuspended in 100 μL of binding buffer (10 mM HEPES, 140 mM NaCl, and 2 mM CaCl_2_; pH 7.4). The cells were then incubated at room temperature for 15 min, and PI was added before flow cytometry or fluorescence microscopy analysis. Apoptosis were determined using a FACS calibur (Becton and Dickson) and analysed using Cell Quest pro software. Images were analyzed using NIS Elements software (Nikon).

### Assessment of cytoplasmic and mitochondrial ROS levels

The levels of cytoplasmic ROS were estimated using the oxidation-sensitive fluorescent dye H_2_DCF-DA (2′,7′-dichlorodihydrofluorescein diacetate; Invitrogen) or the Amplex Red hydrogen peroxide assay kit (Invitrogen). For DCF staining, the cells were loaded with H_2_DCF-DA (100 nM) for 1 h at 37°C and washed once with PBS. After treatment with Khz-cp, ROS levels were analyzed using a flow cytometer (FACSCalibur; Becton Dickinson, San Jose, CA, USA) or a fluorescence microscope (Eclipse 80i; Nikon, Tokyo, Japan). The Amplex Red hydrogen peroxide assay was performed according to the manufacturer’s protocol. In brief, the cells were lysed in 50 μM Amplex Red solution supplemented with 0.1 U/mL horseradish peroxidase and incubated in the dark for 30 min. Fluorescence was measured using a plate reader (Victor 2; Perkin-Elmer Life Sciences, Boston, MA, USA) with an excitation wavelength of 540 nm and an emission wavelength of 590 nm.

Mitochondrial superoxide anion levels were analyzed by staining with MitoSOX™ Red (Invitrogen). Cells were loaded with MitoSOX Red (5 μM) for 30 min at 37°C and then treated with Khz-cp. The fluorescence was then analyzed by flow cytometry or fluorescence microscopy.

### Preparation of subcellular fractions

To prepare the mitochondrial and cytosolic fractions, the cells (1 × 10^7^) were washed once in PBS and disrupted by passing them through a glass homogenizer 80 times in ice-cold isolation buffer (250 mM sucrose, 20 mM HEPES, 10 mM KCl, 1.5 mM MgCl_2,_ 1 mM EGTA, 1 mM EDTA, 1 mM DTT, and 0.1 mM PMSF). Nuclei and non-disrupted cells were removed by centrifugation at 750 × *g* for 20 min at 4°C. The supernatant was further centrifuged at 10,000 × *g* for 15 min at 4°C to obtain a mitochondrion-enriched pellet and a cytoplasm-enriched supernatant.

Membrane and cytosolic fractions were prepared using the Compartmental Protein Extraction kit (Millipore, Temecula, CA, USA) according to the manufacturer’s instructions.

### Ca^2+^ imaging

Digital imaging of the intracellular free Ca^2+^ was performed using the fura-2 AM dye (Invitrogen). When fura-2 binds to Ca^2+^, its maximal absorption wavelength shifts from 363 to 335 nm. SNU-1 cells (1 × 10^4^) were cultured in 35-mm glass-bottomed dishes and loaded with fura-2 AM (2 μM) for 30 min at 37°C. Fluorescence images of fura-2 were digitally captured at excitation wavelengths of 340 and 380 nm and an emission wavelength of 510 nm with an IX70 fluorescence microscope (Olympus, Tokyo, Japan) equipped with a digital cooled charge-coupled device camera. Paired 340/380 ratiometric images were analyzed using the Metafluor software (Molecular Devices, Sunnyvale, CA, USA). Confocal images of intracellular free Ca^2+^ were obtained using the fluo-4 AM Ca^2+^-sensitive fluorescent dye (Invitrogen). The cells were loaded with fluo-4 AM (1 μM) for 30 min at 37°C, and Ca^2+^ imaging was performed using a confocal laser scanning microscope (LSM510; Carl Zeiss, Jena, Germany).

### Transfection of siRNA and plasmids

SNU-1 cells were transfected with siRNA using Lipofectamine 2000 (Invitrogen), as described previously. The coding strand sequences of the siRNA were as follows: 5′-CUG GUA UGA UCC UUC UGA AdTdT-3′ (P381), 5′-GAG GUA UAC ACA UAC UGA dTdT-3′ (Nox2), 5′-CUG UUG UGG ACC CAA UUC AdTdT-3′ (Nox4), and 5′-GUU CAG CGU GUC CGG CGA GdTdT-3′ (GFP). Bcl-2 cDNA was transfected into cells using the Lipofectamine-PLUS reagent (Invitrogen) according to the manufacturer’s instructions. Stably transfected cells were selected using G418 (3 mg/mL).

## Results

### Khz-cp induces apoptosis in transformed cells

Khz-cp is shown in Figure 1A. The aim of the present study was to examine whether Khz-cp causes apoptosis in human cancer cells and, if so, to identify the signaling mechanisms involved. As shown in Figure 1b and c, staining with annexin-V-FITC and PI showed that Khz-cp triggered apoptosis in SNU-1 cells (annexin-V single-positive or annexin-V/PI double-positive cells) as early as 60 min after treatment. As apoptosis progressed, the population of cells stained with annexin-V alone declined, whereas that of cells stained with both annexin-V and PI increased (Figure 1c). The population of cells positive for PI alone may represent necrotic cells. The induction of preferential apoptosis by Khz-cp in transformed cells was then evaluated in a series of cell lines that comprise an *in vivo* lung epithelial carcinogenesis model. BEAS-2B is an immortalized normal human bronchial epithelial cell line, and 1198 and 1170-I are transformed cell lines derived from BEAS-2B cells exposed *in vivo* to beeswax pellets containing cigarette smoke condensate (CSC). The 1799 cell line is a non-transformed line derived from BEAS-2B cells exposed to beeswax alone. Khz-cp induced apoptosis in the transformed 1198 and 1170-I cells but not in non-transformed BEAS-2B and 1799 cells (Figure 1d). These data indicate that Khz-cp induces apoptosis preferentially in cancer cells, which suggests that it has potential as a therapeutic agent against cancer.

### Khz-cp-induced apoptosis is caspase dependent and occurs through a mitochondrial pathway

**Figure 2:**
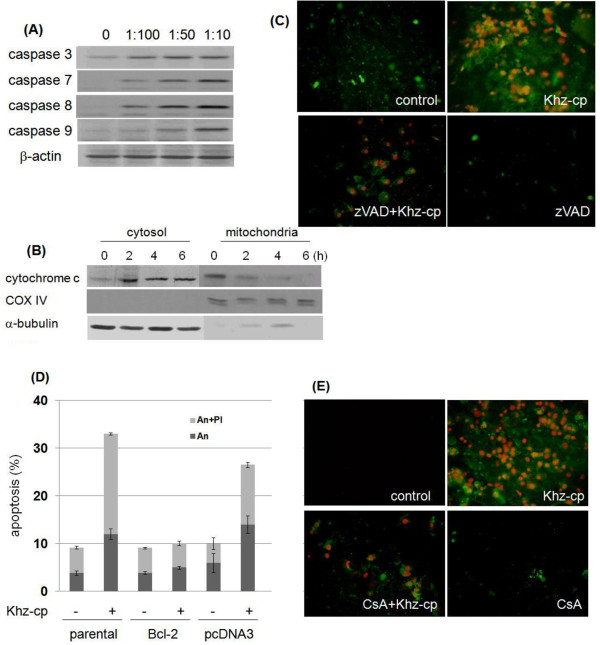
**Khz-cp induces apoptosis through a mitochondrial pathway. (A)** SNU-1 cells were treated with a 1:100 dilution of Khz-cp, and caspase or PARP activation was analyzed by immunoblotting. **(B)** Immunoblot analysis of cytochrome *c* levels in cytosolic and mitochondrial fractions of SNU-1 cells treated with Khz-cp 1 h. **(C)** Cells were pretreated with z-VAD-fmk (20 μM) for 1 h and treated with Khz-cp at the same concentrations used in Figure 2(B). After 1 h, the cells were stained with propidium iodide (PI) and annexin-V-FITC (An) for fluorescent microscopy. **(D)** SNU-1 cells stably transfected with Bcl-2 cDNA or an empty vector (pcDNA3) were treated with Khz-cp for 1 h. Apoptosis was analyzed as in Figure 2(C). **(E)** Cells were pretreated with CsA (10 μM) for 1 h. Khz-cp treatment and apoptosis analysis were performed as in Figure 2(B). After 1 h, the cells were stained with PI and An for fluorescent microscopy.

To determine whether the Khz-cp-induced apoptosis was caspase dependent, caspase activation was analyzed after Khz-cp treatment. Cleavage of caspase 3 and PARP (indicating their activation) increased in SNU-1 cells after Khz-cp treatment (Figure 2a). Moreover, pretreatment of these cells with the pan-caspase inhibitor z-VAD-fmk completely blocked Khz-cp-induced apoptosis (Figure 2b), which indicates that Khz-cp induces caspase-dependent apoptosis.

Natural products or cytotoxic chemicals often induce apoptosis through a mitochondrial pathway; therefore, the release of cytochrome *c* from the mitochondria into the cytosol was analyzed to determine whether Khz-cp-induced apoptosis also occurred *via* a mitochondrial pathway. As shown in Figure 2c, cytochrome *c* levels in the cytosol of SNU-1 cells increased after Khz-cp treatment, whereas cytochrome *c* levels in the mitochondria concurrently decreased, which indicates the release of mitochondrial cytochrome *c*. Ectopic expression of the protective Bcl-2 protein prevented Khz-cp-induced apoptosis in SNU-1 cells (Figure 2d). Furthermore, treatment with CsA (which blocks PTP opening by binding to cyclophilin D) abrogated Khz-cp-induced apoptosis, which suggests that mitochondrial permeability transition is required for apoptosis (Figure 2e).

### Oxidative stress mediates Khz-cp-induced apoptosis

**Figure 3:**
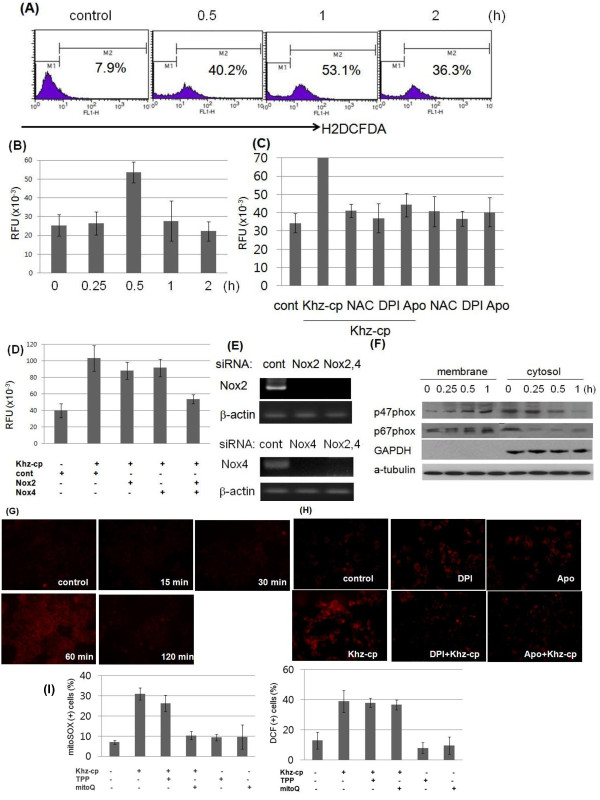
**Khz-cp triggers cytoplasmic and mitochondrial ROS generation. (A)** SNU-1 cells were loaded with H_2_DCF-DA and treated with Khz-cp (diluted 1:100), and the cytoplasmic ROS levels were assessed by flow cytometry. **(B)** Intracellular ROS levels in SNU-1 cells treated with Khz-cp (diluted 1:100) were analyzed using an Amplex Red hydrogen peroxide assay. **(C)** The cells were pretreated with NAC (5 mM), DPI (10 μM), or apocynin (Apo; 300 μM) for 1 h. Intracellular ROS levels in SNU-1 cells were analyzed 30 min after Khz-cp treatment by using the Amplex Red hydrogen peroxide assay. **(D)** SNU-1 cells were transfected with siRNA targeting Nox2 and Nox4. After 48 h, the cells were treated with Khz-cp (1:100, 0.5 h), and ROS generation was measured after 60 min by using the Amplex Red hydrogen peroxide assay. **(E)** Silencing of Nox2 and Nox4 by siRNA transfection was assessed by RT-PCR. **(F)** SNU-1 cells were treated with Khz-cp, and the membrane and cytosol fractions were separated using the Compartmental Protein Extraction kit. The expression of the p47^phox^ and p67^phox^ proteins in the membrane and cytosol fractions was analyzed by immunoblotting. **(G)** SNU-1 cells were loaded with MitoSOX Red for 30 min and treated with Khz-cp. Mitochondrial ROS generation was then assessed by fluorescent microscopy at the indicated time points. **(H)** SNU-1 cells were pretreated with DPI or apocynin for 1 h, and mitochondrial ROS generation was analyzed 60 min after Khz-cp treatment as in (G). **(I)** SNU-1 cells were pretreated with 0.5 μM MitoQ or TPP for 30 min. Right panel: Cytoplasmic ROS generation was measured 30 min after Khz-cp treatment by DCF staining and flow cytometry. Left panel: Mitochondrial ROS generation was assessed 60 min after Khz-cp treatment by MitoSOX Red staining and flow cytometry.

ROS production after Khz-cp treatment was analyzed because oxidative stress is typically involved in apoptosis. Figure 3a and b show that cytoplasmic ROS levels increased 30 min after Khz-cp treatment. Therefore, NADPH oxidase was investigated as a potential source of ROS generation after Khz-cp treatment. Figure 3c shows that DPI (flavoprotein inhibitor) and apocynin (p47^phox^ inhibitor) prevented ROS production. Moreover, silencing of Nox2 and Nox4 by using specific siRNAs almost completely abrogated ROS production (Figure 3d, e). Transfection with Nox2 or Nox4 siRNA alone partially blocked ROS production, which suggests that Nox2 and Nox4 contribute to Khz-cp-induced ROS generation in combination. Activation of NADPH oxidase by Khz-cp was also shown by the translocation of the cytosolic subunits of NADPH oxidase p47^phox^ and p67^phox^ to the cell membrane 15 min after Khz-cp treatment (Figure 3f). Taken together, these data indicate that NADPH oxidase produces ROS upon Khz-cp treatment.Because the mitochondrial respiratory chain is another major source of cellular ROS, mitochondrial ROS production was evaluated using MitoSOX Red staining. Mitochondrial ROS levels in SNU-1 cells increased 60 min after Khz-cp treatment (Figure 3g); therefore, it appears that ROS production by mitochondria occurs later than that by NADPH oxidase. Mitochondrial ROS generation by Khz-cp was prevented by pretreatment with DPI or apocynin, which suggests that NADPH oxidase plays a critical role in mitochondrial ROS production (Figure 3h). Furthermore, the mitochondria-targeting antioxidant MitoQ did not significantly block cytoplasmic ROS generation at a concentration that completely blocked mitochondrial ROS production (Figure 3i). TPP, which is the lipophilic moiety of MitoQ, was used as a control. Taken together, these data show that mitochondrial ROS generation induced by Khz-cp was mediated indirectly by NADPH oxidase-derived ROS.

**Figure 4:**
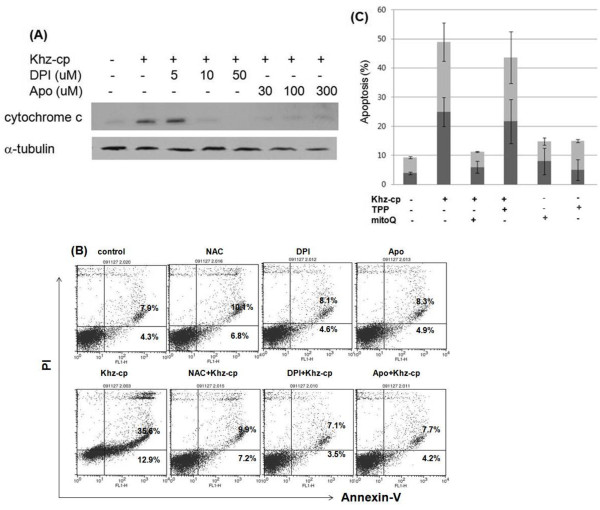
**ROS generation by NADPH oxidase and mitochondria is required for Khz-cp-induced apoptosis. (A)** SNU-1 cells were pretreated with DPI or apocynin for 1 h, and the cytochrome *c* levels in the cytosol were analyzed 2 h after Khz-cp treatment by immunoblot analysis. **(B)** Cells were pretreated with DPI (10 μM), apocynin (300 μM), or NAC (5 mM) for 30 min, and apoptosis was examined by annexin-V-FITC (An) and PI staining followed by flow cytometric analysis. **(C)** SNU-1 cells were pretreated with 0.5 μM MitoQ or TPP for 30 min, and apoptosis was analyzed as in Figure 1(C).

We further examined whether ROS generation through NADPH oxidase and/or mitochondria was necessary for Khz-cp-induced apoptosis. Pretreatment of cells with DPI or apocynin suppressed mitochondrial cytochrome *c* release and apoptosis induced by Khz-cp treatment, which indicates that ROS generation through NADPH oxidase was required for the induction of apoptosis (Figure 4a, b). Furthermore, pretreatment with MitoQ, but not TPP, prevented Khz-cp-induced apoptosis, which suggests that mitochondrial ROS generation was also necessary for the induction of apoptosis (Figure 4c). Therefore, NADPH oxidase-derived ROS appear to trigger apoptosis *via* mitochondrial ROS generation.

### Khz-cp induces a rapid and sustained increase in [Ca^2+^]_*i*_

**Figure 5:**
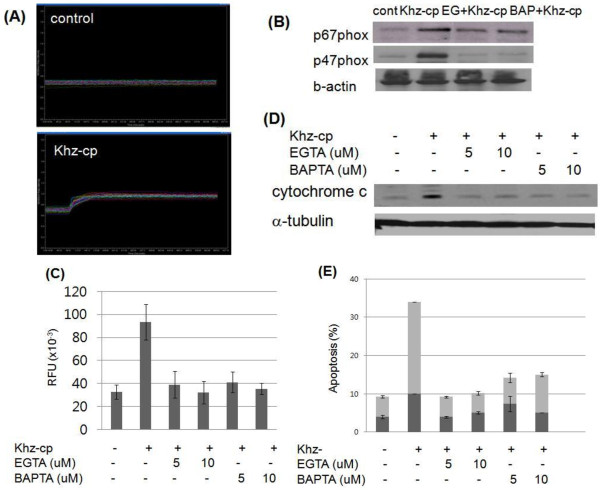
**An increase in [Ca**^**2+**^**]**_***i***_**is necessary for Khz-cp-induced ROS generation and apoptosis. (A)** SNU-1 cells were loaded with fura-2 AM for 30 min, and changes in [Ca^2+^]_*i*_ after Khz-cp treatment (50 μg/ml) were analyzed by digital imaging microscopy. **(B)** SNU-1 cells were pretreated with EGTA (10 μM) or BAPTA-AM (10 μM) for 30 min, and membrane fractions were prepared for immunoblot analysis 12 h after Khz-cp treatment. (C-E) Cells were pretreated with EGTA or BAPTA-AM for 30 min and treated with Khz-cp (1:100 dilution). After 12 h, ROS generation was measured in SNU-1 cells by an Amplex Red hydrogen peroxide assay. The data are presented as the mean ± SD values **(C)**. Cytosolic cytochrome *c* levels were analyzed in SNU-1 cells by immunoblot analysis **(D)**. Apoptosis was analyzed 12 h after Khz-cp treatment **(E)**.

Because increased [Ca^2+^]_*i*_ has been widely implicated in mitochondria-mediated apoptosis, we evaluated whether Khz-cp affected [Ca^2+^]_*i*_ and, if so, whether it played a role in the activation of NADPH oxidase and ROS generation. Figure 5a shows that Khz-cp induced an immediate and sustained increase in [Ca^2+^]_*i*_ in SNU-1 cells. The effects of the increase in [Ca^2+^]_*i*_ on the activation of NADPH oxidase were then examined. EGTA and BAPTA-AM, which are extracellular and intracellular Ca^2+^ chelators, respectively, prevented NADPH oxidase activation as measured by the membrane translocation of the p47^phox^ and p67^phox^ subunits (Figure 5b). Furthermore, both of these chelators inhibited ROS generation (Figure 5c) and blocked Khz-cp-induced mitochondrial cytochrome *c* release and apoptosis (Figure 5d, e). These results indicate that Khz-cp triggers a rapid and sustained increase in [Ca^2+^]_*i*_ that activates NADPH oxidase to induce ROS generation and, finally, apoptosis.

### P38 mediates Ca^2+^-dependent activation of NADPH oxidase

**Figure 6:**
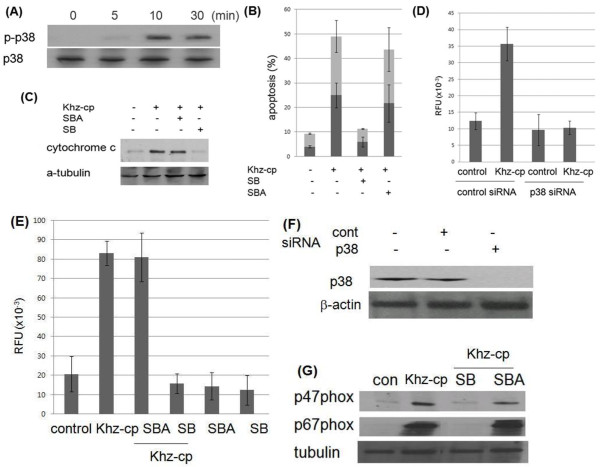
**P38 mediates the increase in [Ca**^**2+**^**]**_***i***_**to induce NADPH oxidase-derived ROS generation. (A)** Activation of P38 was analyzed in Khz-cp-treated SNU-1 cells by immunoblot analysis of phosphorylated P38. **(B)** Cells were pretreated with 20 μM SB203580 (SB) and SB202474 (SBA) for 1 h. Apoptosis was analyzed 1 h after Khz-cp treatment as in Figure 2(C). **(C)** SNU-1 cells were pretreated with P38 inhibitors as in (B), and cytochrome c levels in the cytosol were analyzed by immunoblotting 2 h after Khz-cp treatment. **(D, E)** SNU-1 cells were transfected with control or P38-1 siRNA. After 48 h, ROS generation was analyzed 30 min after Khz-cp treatment using the Amplex Red hydrogen peroxide assay. The data represent the mean ± SD values **(D)**. P38 protein expression was analyzed by immunoblotting 48 h after transfection **(E). (F, G)** SNU-1 cells were pretreated with 20 μM SB203580 (SB) and SB202474 (SBA) for 1 h, and ROS generation induced by Khz-cp treatment was measured using the Amplex Red hydrogen peroxide assay. The data represent the mean ± SD values **(F)**. The levels of p47^phox^ and p67^phox^ proteins in the membrane fractions of SNU-1 cells were analyzed by immunoblotting 15 min after Khz-cp treatment **(G)**.

The mitogen-activated protein kinase P38 is activated in response to oxidative stress [32,33]. Because Khz-cp induced ROS generation, the activation of P38 and its possible role in Khz-cp-induced apoptosis were examined. As shown in Figure 6a, P38 was activated as early as 15 min after Khz-cp treatment. Considering that ROS generation was observed 30 min after Khz-cp treatment (Figure 4b), it is unlikely that the activation of P38 was caused by oxidative stress. Nonetheless, P38 activity was required for the induction of apoptosis because a chemical inhibitor of P38 (SB203580) blocked mitochondrial cytochrome *c* release and apoptosis induced by Khz-cp treatment, but not an inactive analog of SB202474 (Figure 6b, c). Inhibition of P38 by siRNA transfection or pretreatment with chemical inhibitors suppressed Khz-cp-induced ROS production, which indicates that the activation of P38 caused ROS generation rather than the opposite (Figure 6d–f). In agreement with these findings, pretreatment of SNU-1 cells with SB203580 also suppressed the activation of NADPH oxidase, as assessed by the membrane translocation of p47^phox^ and p67^phox^ (Figure 6g). Therefore, we examined whether P38 activation was dependent on [Ca^2+^]_*i*_. Collectively, these results strongly indicate that P38 is activated by Khz-cp *via* an increase in [Ca^2+^]_*i*,_ thereby triggering ROS generation by NADPH oxidase and the induction of apoptosis.

## Discussion

In the present study, Khz-cp triggered caspase-dependent and mitochondria-mediated apoptotic death in human cancer cells. Khz-cp-induced apoptosis was selective for transformed cells, and Khz-cp only minimally affected non-transformed cells, which suggests that it is a potential anticancer therapeutic agent. Intrinsic oxidative stress in transformed cells may render them more susceptible to apoptosis induced by Khz-cp. Alternatively, cancer cells may have defective restoration of glutathione (GSH) reserves. However, the GSH levels in tumor tissues are higher than those in normal tissues [34], and depleting GSH reserves often sensitizes cancer cells to ROS-induced cell death, which suggests that the GSH present in cancer cells protects them from oxidative stress.

Khz-cp-induced apoptosis was observed to be caspase dependent, as indicated by the activation of caspases and inhibition of apoptosis by pretreatment with the pan-caspase inhibitor z-VAD-fmk (Figure 2a, b). The involvement of a mitochondria-mediated pathway was confirmed by the release of mitochondrial cytochrome *c*, and inhibition of apoptosis was confirmed by the overexpression of Bcl-2 (Figure 2c, d). Oxidative stress is associated with apoptotic and non-apoptotic cell death, although pro-oxidative conditions are not a prerequisite for apoptosis [35]. The present study shows that the induction of apoptosis by Khz-cp required ROS generation by both NADPH oxidase and mitochondria. Several studies have implicated ROS generated from mitochondria in the induction of apoptosis; however, the results of the present study indicate that NADPH oxidase-derived ROS were critical for Khz-cp-induced apoptosis (Figure 4a, b). Although mitochondrial ROS generation was also necessary for Khz-cp-induced apoptosis (Figure 4c), its effect was secondary to that of the initial ROS production by NADPH oxidase because the generation of mitochondrial ROS occurred after that of cytoplasmic ROS and was prevented by pretreatment with NADPH oxidase inhibitors (Figure 3g, h). Of the 7 members of the human NADPH oxidase family, Nox2 and Nox4 were found to be responsible for the ROS generation induced by Khz-cp treatment in SNU-1 cells (Figure 3d, e).

It is widely accepted that calcium signaling plays an important role in apoptosis [29,36]. Cross-talk between ROS and calcium signaling pathways may lead to synergistic effects on mitochondrial permeabilization and cell death. The present study showed that an increase in the [Ca^2+^]_*i*_ induced by Khz-cp treatment resulted in ROS generation by NADPH oxidase. The [Ca^2+^]_*i*_ increased before the generation of ROS, and Ca^2+^ chelators such as EGTA and BAPTA-AM abrogated the activation of NADPH oxidase and ROS generation (Figure 5b, c). However, the inhibitors of NADPH oxidase did not affect the increase in [Ca^2+^]_i_ induced by Khz-cp treatment (data not shown). The activation of NADPH oxidase by elevated [Ca^2+^]_*i*_ in neutrophils is well known and has also been reported for other cell types [37,38].

P38 was identified as the mediator of Ca^2+^-dependent NADPH oxidase activation. P38 was activated within 15 min of Khz-cp treatment in a Ca^2+^-dependent manner (Figure 6a) Furthermore, treatment with P38 siRNA or chemical inhibitors prevented Khz-cp-induced ROS generation (Figure 6d–g). Most studies place ROS upstream of P38 activation [39,40] however, in this study, ROS generation by NADPH oxidase was found to be mediated by the activation of P38.

## Conclusions

**Figure 7:**
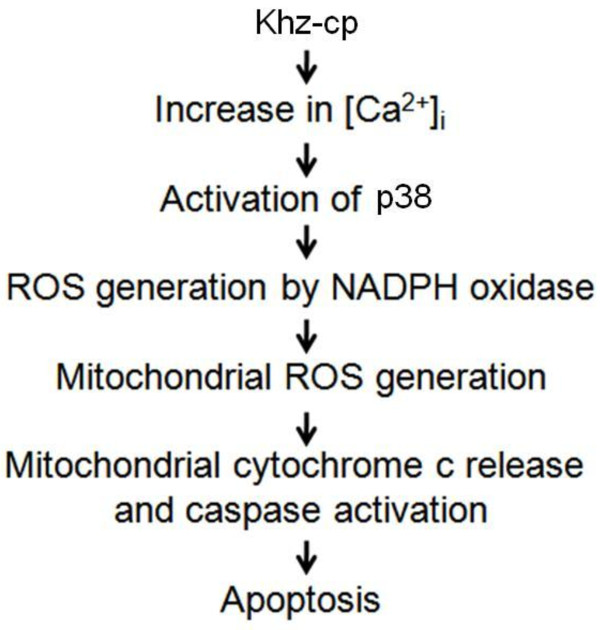
Summary of the signaling events leading to Khz-cp-induced apoptosis.

In this study, we investigated that Khz induces apoptosis by increasing intracellular calcium levels and activating JNK and NADPH oxidase-dependent generation of ROS [12]. We showed that Khz-cp induces mitochondrion-mediated apoptosis preferentially in transformed cells, and the signaling pathway of Khz-cp-induced apoptosis involves an increase in [Ca^2+^]_*i*_, activation of P38, and ROS generation through NADPH oxidase and mitochondria (Figure 7).

## Competing interests

The authors declare that they have no competing interests.

## Authors’ contributions

KTH, JSK, ZHK, RBH,YLC carried out the study and prepared the manuscript. KTH and RSW critically revised manuscript. All authors have read and approved the manuscript for publication.

## Pre-publication history

The pre-publication history for this paper can be accessed here:

http://www.biomedcentral.com/1472-6882/14/236/prepub

## References

[B1] Debatin KM Apoptosis pathways in cancer and cancer therapy Cancer Immunol Immunother 2004 53 153 159 14749900 10.1007/s00262-003-0474-8 PMC11032870

[B2] Arends MJ Wyllie AH Apoptosis: mechanisms and roles in pathology Int Rev Exp Pathol 1991 32 223 254 1677933 10.1016/b978-0-12-364932-4.50010-1

[B3] Mesner PW Jr Budihardjo II Kaufmann SH Chemotherapy-induced apoptosis Adv Pharmacol 1997 41 461 499 9204156 10.1016/s1054-3589(08)61069-8

[B4] Palombo JD Ganguly A Bistrian BR Menard MP The antiproliferative effects of biologically active isomers of conjugated linoleic acid on human colorectal and prostatic cancer cells Cancer Lett 2002 177 163 172 11825663 10.1016/s0304-3835(01)00796-0

[B5] Wasser SP Weis AL Therapeutic effects of substances occurring in higher Basidiomycetes mushrooms: a modern perspective Crit Rev Immunol 1999 19 65 96 9987601

[B6] Jiang J Sliva D Novel medicinal mushroom blend suppresses growth and invasiveness of human breast cancer cells Int J Oncol 2010 37 6 1529 1536 21042722 10.3892/ijo_00000806

[B7] Lin ZB Zhang HN Anti-tumor and immunoregulatory activities of Ganoderma lucidum and its possible mechanisms Acta Pharmacol Sin 2004 25 1387 1395 15525457

[B8] Sanodiya BS Thakur GS Baghel RK Prasad GB Bisen PS Ganoderma lucidum: a potent pharmacological macrofungus Curr Pharm Biotechnol 2009 10 717 742 19939212 10.2174/138920109789978757

[B9] Gao Y Gao H Chan E Tang W Xu A Yang H Huang M Lan J Li X Xu C Zhou S Duan W Antitumor activity and underlying mechanisms of ganopoly, the refined polysaccarides extracted from Ganoderma lucidum, in mice Immunol Investig 2005 34 171 198 15921158

[B10] Yue GG Fung KP Tse GM Leung PC Lau CB Comparative studies of various ganoderma species and their different parts with regard to antitumor and immunomodulating activities in vitro J Altern Complement Med 2006 12 777 789 17034284 10.1089/acm.2006.12.777

[B11] Zhao YY Chao X Zhang Y Lin RC Sun WJ Cytotoxic steroids from Polyporus umbellatus Planta Med 2010 76 15 1755 1758 20458671 10.1055/s-0030-1249926

[B12] Kim TH Kim JS Kim ZH Huang RB Wang RS Khz (fusion of ganoderma lucidum and polyporus umbellatus mycelia) induces apoptosis by increasing intracellular calcium levels and activating JNK and NADPH oxidase-dependent generation of reactive oxygen species PLoS One 2012 7 10 e46208 23056263 10.1371/journal.pone.0046208 PMC3466234

[B13] Kim TH Kim J Kim Z Huang RB Wang RS Khz (Fusion of Ganoderma lucidum and Polyporus umbellatus Mycelia) Induces Apoptosis in A549 Human Lung Cancer Cells by Generating Reactive Oxygen Species and Decreasing the Mitochondrial Membrane Potential Food Sci 2014 23 3 859 864

[B14] Ryter SW Kim HP Hoetzel A Park JW Nakahira K Wang X Choi AM Mechanisms of cell death in oxidative stress Antioxid Redox Signal 2007 9 49 89 17115887 10.1089/ars.2007.9.49

[B15] Buttke TM Sandstrom PA Oxidative stress as a mediator of apoptosis Immunol Today 1994 15 7 10 8136014 10.1016/0167-5699(94)90018-3

[B16] Jacobson MD Reactive oxygen species and programmed cell death Trends Biochem Sci 1996 21 83 86 8882579

[B17] Miyajima A Nakashima J Yoshioka K Role of reactive oxygen species in cis-dichlorodiammineplatium-induced cytotoxicity on bladder cancer cells Br J Cancer 1997 76 206 210 9231920 10.1038/bjc.1997.363 PMC2223948

[B18] Zhou Y Hileman EO Plunkett W Keating MJ Huang P Free radical stress in chronic lymphocytic leukemia cells and its role in cellular sensitivity to ROS-generating anticancer agents Blood 2003 101 4098 4104 12531810 10.1182/blood-2002-08-2512

[B19] Schulze-Osthoff K Bakker AC Vanhaesebroeck B Beyaert R Jacob WA Fiers W Inhibition of mitochondrial respiration: a novel strategy to enhance drug-induced apoptosis in human leukemia cells by a reactive oxygen species-mediated mechanism J Biol Chem 2003 278 37832 37839 12853461 10.1074/jbc.M301546200

[B20] Quillet-Mary A Jaffrézou JP Mansat V Bordier C Naval J Laurent G Cytotoxic activity of tumor necrosis factor is mediated by early damage of mitochondrial functions. Evidence for the involvement of mitochondrial radical generation J Biol Chem 1992 267 5317 5323 1312087

[B21] Fleury C Mignotte B Vayssiere JL Implication of mitochondrial hydrogen peroxide generation in ceramide-induced apoptosis J Biol Chem 1997 272 21388 21395 9261153 10.1074/jbc.272.34.21388

[B22] Fleury C Mignotte B Vayssière JL Mitochondrial reactive oxygen species in cell death signaling Biochimie 2002 84 131 141 12022944 10.1016/s0300-9084(02)01369-x

[B23] Ott M Gogvadze V Orrenius S Zhivotovsky B Mitochondria, oxidative stress and cell death Apoptosis 2007 12 913 922 17453160 10.1007/s10495-007-0756-2

[B24] Hiraoka W Vazquez N Nieves-Neira W Chanock SJ Pommier Y Role of oxygen radicals generated by NADPH oxidase in apoptosis induced in human leukemia cells J Clin Invest 1998 102 1961 1968 9835621 10.1172/JCI3437 PMC509148

[B25] Qin F Patel R Yan C Liu W NADPH oxidase is involved in angiotensin II-induced apoptosis in H9C2 cardiac muscle cells: effects of apocynin Free Radic Biol Med 2006 40 236 246 16413406 10.1016/j.freeradbiomed.2005.08.010

[B26] Brennan AM Suh SW Won SJ Narasimhan P Kauppinen TM Lee H Edling Y Chan PH Swanson RA NADPH oxidase is the primary source of superoxide induced by NMDA receptor activation Nat Neurosci 2009 12 857 863 19503084 10.1038/nn.2334 PMC2746760

[B27] Nicotera P Orrenius S The role of calcium in apoptosis Cell Calcium 1998 23 173 180 9601613 10.1016/s0143-4160(98)90116-6

[B28] Granfeldt D Samuelsson M Karlsson A Capacitative Ca2+ influx and activation of the neutrophil respiratory burst. Different regulation of plasma membrane- and granule-localized NADPH-oxidase J Leukoc Biol 2002 71 611 617 11927647

[B29] Wang G Anrather J Glass MJ Tarsitano MJ Zhou P Frys KA Pickel VM Iadecola C Nox2, Ca2+, and protein kinase C play a role in angiotensin II-induced free radical production in nucleus tractus solitaries Hypertension 2006 48 482 489 16894058 10.1161/01.HYP.0000236647.55200.07

[B30] Kim JE Koo KH Kim YH Sohn J Park YG Identification of potential lung cancer biomarkers using an in vitro carcinogenesis model Exp Mol Med 2008 40 709 720 19116456 10.3858/emm.2008.40.6.709 PMC2679342

[B31] Klein-Szanto AJ Iizasa T Momiki S Garcia-Palazzo I Caamano J Metcalf R Welsh J Harris CC A tobacco-specific N-nitrosamine or cigarette smoke condensate causes neoplastic transformation of xenotransplanted human bronchial epithelial cells Proc Natl Acad Sci U S A 1992 89 6693 6697 1323115 10.1073/pnas.89.15.6693 PMC49569

[B32] Kelso GF Porteous CM Coulter CV Hughes G Porteous WK Ledgerwood EC Smith RA Murphy MP Selective targeting of a redox-active ubiquinone to mitochondria within cells: antioxidant and antiapoptotic properties J Biol Chem 2001 276 4588 4596 11092892 10.1074/jbc.M009093200

[B33] Matsuzawa A Ichijo H Redox control of cell fate by MAP kinase: physiological roles of ASK1-MAP kinase pathway in stress signaling Biochim Biophys Acta 2008 1780 1325 1336 18206122 10.1016/j.bbagen.2007.12.011

[B34] Kuppusamy P Li H Ilangovan G Cardounel AJ Zweier JL Yamada K Krishna MC Mitchell JB Noninvasive imaging of tumor redox status and its modification by tissue glutathione levels Cancer Res 2002 62 307 312 11782393

[B35] Jacobson MD Raff MC Programmed cell death and Bcl-2 protection in very low oxygen Nature 1995 374 814 816 7536895 10.1038/374814a0

[B36] Scorrano L Oakes SA Opferman JT Cheng EH Sorcinelli MD Pozzan T Korsmeyer SJ BAX and BAK regulation of endoplasmic reticulum Ca2+: a control point for apoptosis Science 2003 300 135 139 12624178 10.1126/science.1081208

[B37] Yu JH Lim JW Kim KH Morio T Kim H NADPH oxidase and apoptosis in cerulein-stimulated pancreatic acinar AR42J cells Free Radic Biol Med 2005 39 590 602 16085178 10.1016/j.freeradbiomed.2005.04.019

[B38] Gandhi S Wood-Kaczmar A Yao Z Plun-Favreau H Deas E Klupsch K Downward J Latchman DS Tabrizi SJ Wood NW Duchen MR Abramov AY PINK1-associated Parkinson’s disease is caused by neuronal vulnerability to calcium-induced cell death Mol Cell 2009 33 627 638 19285945 10.1016/j.molcel.2009.02.013 PMC2724101

[B39] Benhar M Dalyot I Engelberg D Levitzki A Enhanced ROS production in oncogenically transformed cells potentiates c-Jun N-terminal kinase and p38 mitogen-activated protein kinase activation and sensitization to genotoxic stress Mol Cell Biol 2001 21 6913 6926 11564875 10.1128/MCB.21.20.6913-6926.2001 PMC99868

[B40] Saeki K Kobayashi N Inazawa Y Zhang H Nishitoh H Ichijo H Saeki K Isemura M Yuo A Oxidation-triggered c-Jun N-terminal kinase (JNK) and p38 mitogen-activated protein (MAP) kinase pathways for apoptosis in human leukaemic cells stimulated by epigallocatechin-3-gallate (EGCG): a distinct pathway from those of chemically induced and receptor-mediated apoptosis Biochem J 2002 368 705 720 12206715 10.1042/BJ20020101 PMC1223028

